# Is More Energy Reliably Better in Energy‐Based Aesthetic Practice?

**DOI:** 10.1111/jocd.71084

**Published:** 2026-07-31

**Authors:** Kyu‐Ho Yi

**Affiliations:** ^1^ You and I Clinic Seoul Korea

In energy‐based aesthetic practice, patients may assume that a stronger energy setting will produce better tightening or faster collagen remodeling. This assumption is biologically incomplete. More energy is not reliably better; the clinically relevant goal is an anatomically appropriate thermal dose that is sufficient to induce tissue remodeling while remaining within device‐specific and tissue‐specific safety limits. The clinician's task is to match energy delivery, electrode configuration, treatment depth, pass number, and tissue response to the target indication while avoiding unnecessary injury to skin, fat, vessels, nerves, bone, and sensitive aesthetic subunits.

In this commentary, thermal dose refers to the cumulative biological effect of heat exposure, determined by temperature, exposure time, tissue depth, heat distribution, cooling, impedance, hydration, and local tissue characteristics. It is therefore different from the displayed device output or peak energy setting. This commentary primarily focuses on RF‐based aesthetic procedures, including monopolar RF (radiofrequency), bipolar RF, and RF microneedling. HIFU/MFU and lasers are mentioned only as related thermal technologies because their mechanisms of energy delivery, depth targeting, tissue interaction, and safety profiles differ substantially. Energy settings across RF, HIFU/MFU, RF microneedling, and lasers should not be directly compared.

Displayed energy settings do not directly equal biological dose, because tissue response is influenced by impedance, hydration, electrode–tissue contact, cooling, tissue thickness, local perfusion, treatment duration, and anatomical location. Collagen remodeling occurs within a therapeutic window: Insufficient heating may fail to stimulate remodeling, whereas excessive exposure may cause pain, inflammation, fat injury, burns, scarring, prolonged edema, dysesthesia, or other complications. Therefore, stronger settings are not inherently superior and should be used only when the target tissue, treatment endpoint, device labeling, and patient‐specific anatomy justify them.

Immediate swelling, heat, pain, erythema, or transient tightness should not be interpreted as proof of effective collagen remodeling because true extracellular matrix remodeling evolves over weeks to months.

The dose–response relationship is also tissue‐specific and indication‐specific. Dermal tightening, scar remodeling, sebaceous modulation, adipose heating, and lifting‐oriented treatments require different depths, degrees of heating, and safety margins. A setting appropriate for thicker submental tissue may be excessive in the infraorbital cheek, temple, mandibular border, or other thin‐tissue regions. RF‐based treatment should therefore be practiced as anatomical dose prescription rather than parameter escalation.

Thermal dose depends on both temperature and exposure time. Connective‐tissue and energy studies show that collagen contraction, heat‐shock protein activation, procollagen expression, denaturation, and necrosis occur across different thermal ranges rather than at one universal endpoint [1, 2, 3, 4]. Thermal safety should also be understood through a time–temperature framework. Cutaneous thermal pain is commonly encountered around 43°C, which can become a practical limiting factor during aesthetic remodeling procedures [5, 6]. Tissue injury depends not only on peak temperature but also on exposure duration, tissue type, perfusion, cooling, and depth of heat deposition. Concepts such as CEM43 and Arrhenius‐based thermal injury models illustrate that cumulative heat exposure, rather than a single energy number, determines biological risk [7, 8, 9, 10]. Thus, a moderate temperature maintained predictably may be safer and biologically more useful than a brief high‐temperature spike. Practical time–temperature concepts relevant to aesthetic collagen remodeling and thermal safety are summarized in Table 1.

**TABLE 1:** Practical thermal‐temperature concepts for aesthetic collagen remodeling and safety.

Approximate thermal range or endpoint	Biological meaning	Clinical interpretation for aesthetic practice
< 40°C	Mild warming; usually below meaningful collagen denaturation threshold.	May feel comfortable but may be insufficient for remodeling unless exposure time or treatment repetition is adequate.
40°C–45°C	Stress range associated with heat‐shock response and early protein/membrane effects.	Often relevant for controlled RF heating and nonablative stimulation; not automatically visible as dramatic erythema.
~43°C for several min	Lower‐temperature prolonged exposure may support safer collagen remodeling compared with brief excessive heat.	Explains why gradual heating and repeated passes can be clinically effective without extreme pain.
~45°C–50°C	Useful nonablative heating range in many RF concepts, depending on depth and time.	Requires monitoring of patient heat feedback and skin surface safety; excessive pass stacking can still overheat tissue.
~57°C–61°C	Collagen contraction/thermal denaturation range frequently cited for immediate connective‐tissue effects.	May contribute to immediate tightening but also narrows the safety margin near nerves, fat, and thin tissue.
> 60°C	Protein denaturation and increasing risk of necrosis or coagulative injury depending on exposure time.	Appropriate only when focal coagulation is intended and precisely located, as in fractional or focused technologies.
Very high temperatures or short intense spikes	Rapid structural change can occur with very short exposure times.	High‐energy spikes may produce injury without better organized neocollagenesis; avoid treating pain as an efficacy endpoint.

The energy based ligamentous study by Oh et al. provides a useful mechanistic example [1]. In senescent fibroblasts and UV‐induced senescent rat facial ligaments, a 42 W setting produced more favorable HSP70‐mediated remodeling and collagen type I/III restoration than a 73 W setting. The lower‐power condition corresponded to an intradermal temperature of approximately 44.8°C, whereas the higher setting exceeded 59°C [1]. However, this study should be interpreted as preclinical mechanistic evidence rather than direct clinical proof. Because the experiment was performed in cellular and animal models, its findings cannot be directly extrapolated to human aesthetic RF treatment settings. The study supports the concept that a biological response may plateau or become unfavorable with excessive heating, but it does not establish a universal optimal wattage for clinical practice. A comparison of the biological and thermal responses observed under the 42 W and 73 W conditions in this animal model is provided in Table 2.

**TABLE 2:** Why the 42 W condition was more favorable than 73 W in the RF facial‐ligament animal model.

Parameter	42 W RF condition	73 W RF condition	Clinical interpretation
Approximate tissue temperature	About 44.8°C in preliminary/in vivo rationale	Above 59°C in preliminary/in vivo rationale	Higher power increased heat but did not improve the overall molecular remodeling profile.
Cumulative fluence for 10 shots	Approximately 213 J/cm^2^	Approximately 370 J/cm^2^	The higher fluence created a larger thermal load, not necessarily a better regenerative signal.
HSP70 expression	Higher and more favorable	Increased but less favorable than 42 W	Moderate thermal stress may better support HSP70‐mediated remodeling.
HSP70‐IKKγ binding	Stronger interaction	Weaker than 42 W	Supports lower NF‐κB activity through HSP70‐dependent regulation.
NF‐κB pathway	Greater reduction in pIκBα/IκBα ratio and nuclear NF‐κB	Reduced compared with senescent tissue but less than 42 W	Excessive heating may not proportionally suppress inflammatory degradation pathways.
MMPs and SMAD7	Greater reduction of MMP1/2/3/9 and SMAD7	Reduced but less favorable	Better balance between decreased degradation and improved collagen synthesis signaling at 42 W.
Collagen type I/III ratio and fiber structure	Greater restoration of collagen I/III balance, density, and bundle diameter	Improved versus senescent tissue but less favorable than 42 W	The stronger setting was not the better collagenesis setting in this model.
Take‐home message	Biologically optimized lower thermal dose	Higher thermal stress with less favorable remodeling	Energy should be titrated to tissue response, not maximized.

This does not mean that lower energy is always superior. Rather, more energy is not reliably better. The optimal setting is the device‐specific and indication‐specific dose that reaches the intended tissue layer and thermal endpoint while preserving surrounding anatomy.

The evidentiary basis for this commentary should be interpreted in three levels. First, experimental evidence, including cellular, connective‐tissue, animal, and thermal injury models, supports the principle that biological response depends on cumulative heat exposure rather than displayed power alone [1, 2, 3, 4, 7, 8, 9, 10]. Second, clinical studies and safety reports provide practical information on treatment response, tolerability, and adverse events, but they often remain device‐specific and indication‐specific [11, 12, 13, 14, 15, 16, 17, 18, 19, 20, 21]. Third, expert clinical judgment is required to integrate anatomy, tissue thickness, skin type, age, prior procedures, patient tolerance, and device labeling into individualized treatment planning. These categories should not be interpreted as equivalent levels of evidence.

Although this commentary focuses on RF‐based treatment, related thermal technologies illustrate why energy settings cannot be generalized across platforms. HIFU/MFU create discrete thermal injury zones at selected depths, whereas fractional lasers create microscopic columns of photothermal injury and RF microneedling delivers electrical energy through insulated or noninsulated needles [11, 12, 13, 19, 20, 21]. These mechanisms differ substantially from conventional RF heating. Therefore, the principle of appropriate thermal dose may be shared, but numerical energy settings, endpoints, and risk profiles should not be transferred directly between platforms.

## 1 Clinical Safety and Device‐Specific Boundaries

Device labeling and regulatory clearance define the intended use, recommended treatment parameters, contraindications, warnings, and safety boundaries for each device. In clinical practice, exceeding manufacturer‐recommended parameters or using settings outside the labeled indication may create safety and medicolegal concerns. Regulatory documents, including FDA 510(k) summaries or PMA materials when applicable, provide important safety context, although they do not replace clinical judgment or individualized patient assessment. A 510(k) clearance is based on substantial equivalence to a legally marketed device, whereas PMA review evaluates safety and effectiveness for higher‐risk devices under intended conditions of use [22, 23].

Nerve safety should be discussed cautiously because available reports of nerve‐related events after thermal aesthetic procedures are largely case histories or case series and do not establish a precise dose–response relationship between device output and nerve injury [14, 15, 16, 17, 18]. Although many published reports involve HIFU/MFU rather than RF, they remain clinically relevant as cautionary examples showing that thermal energy delivered near vulnerable anatomical pathways may produce uncommon but meaningful neurologic symptoms, including numbness, dysesthesia, brow or perioral weakness, and transient facial nerve branch paresis [14, 15, 16, 17, 18]. Even when temporary, such symptoms may be distressing and should be addressed during consent. Key facial nerve risk zones, potential clinical manifestations, mechanisms of injury, and risk‐reduction strategies during HIFU/MFU treatment are summarized in Table 3.

**TABLE 3:** Nerve‐related risk considerations during HIFU/MFU treatment of the face.

Structure or risk zone	Possible clinical problem	Why excessive or misplaced energy matters	Risk‐reduction strategy
Marginal mandibular branch; mandibular border, pre‐jowl, lower perioral region	Lower‐lip asymmetry, weak depression of lower lip, asymmetric smile, oral incompetence	The branch may become superficial near the facial vessels and lower mandible; focal heat or edema can cause neuropraxia.	Avoid direct line stacking over the mandibular border; reduce energy/depth in thin tissue; do not chase pain; document dynamic movement.
Temporal/frontal branch; temple and lateral brow	Brow or frontalis weakness, eyebrow asymmetry	Thin soft tissue and superficial nerve course can bring motor branches close to the focal zone.	Use conservative settings, avoid aggressive deep passes over thin temple tissue, and maintain proper coupling.
Buccal/zygomatic branches; preauricular and midface zones	Smile asymmetry or cheek movement disturbance	High focal energy near superficial motor branches or parotid‐masseteric fascia can irritate nerves.	Map the midface, avoid repeated high‐density lines in one narrow zone, and stop for twitching or unusual focal pain.
Mental, infraorbital, and sensory branches	Numbness, tingling, dysesthesia, altered sensation	Sensory nerves may be irritated by heat, edema, or treatment near foramina.	Avoid foraminal zones, use anatomical landmarks and ultrasound when available, and follow persistent symptoms.
Bone‐adjacent areas such as mandibular edge, zygoma, orbital rim	Sharp pain, focal inflammation, possible adjacent nerve irritation	Thin soft tissue can place the focal zone near periosteum or reflected heat‐sensitive structures.	Confirm tissue thickness; omit inappropriate deep cartridges; avoid transpalpebral or globe‐directed treatment.

The marginal mandibular branch remains clinically relevant in lower‐face treatment because it may travel near the mandibular border, pre‐jowl sulcus, and thin perioral lower face [24, 25]. Facial nerve branching patterns and soft‐tissue thickness vary between individuals, which reinforces the importance of anatomical treatment planning rather than reliance on a fixed energy setting. Irritation or neuropraxia may present as lower‐lip asymmetry, difficulty depressing the lower lip, asymmetric smile, drooling, or oral incompetence. Symptoms may appear during treatment or within hours to the following day as inflammation or thermal neurapraxia evolves.

The mechanism, when neurologic symptoms occur, is most plausibly transient nerve irritation, inflammation, or focal thermal neurapraxia rather than transection. Although available reports do not prove a linear dose–response relationship, excessive thermal exposure, inappropriate depth, repeated pulses over narrow danger zones, treatment over bony edges, or delivery near vulnerable anatomical pathways may plausibly contribute to nerve irritation. Lower‐face treatment should therefore be planned according to tissue thickness and anatomy, and treatment should stop immediately if electric pain, twitching, focal pain, numbness, or asymmetry occurs.

Patient‐specific planning should also consider small facial frame, thin lower‐face soft tissue, age‐related volume loss, prior slimming procedures, prior surgery, weight loss, previous aggressive energy treatments, and anatomical variation. In these settings, the protective soft‐tissue buffer may be reduced, and conservative parameter selection may be appropriate.

Suspected marginal mandibular neuropraxia should be documented with focused facial nerve examination, photographs or video of dynamic movement, and appropriate follow‐up. Persistent or severe motor findings warrant referral to a neurologist, facial nerve specialist, plastic surgeon, or otolaryngologist.

## 2 Practical Interpretation

Thermal injury can also affect non‐neural structures. Excessive RF heating may worsen volume loss by injuring adipose tissue, especially in thin patients or those with facial deflation. High energy near bone may cause disproportionate pain, and periorbital treatment requires strict avoidance of unsafe globe‐directed or transpalpebral delivery when such use is not specifically indicated or labeled. The target tissue, anatomical plane, and device‐specific safety boundary must therefore be defined before the setting is chosen.

Related technologies such as fractional lasers also demonstrate the broader principle that excessive photothermal injury may increase prolonged erythema, dyspigmentation, infection, or scarring [19, 20, 21]. However, laser fluence and density should not be directly compared with RF power settings because the mechanisms and tissue interactions differ.

Patient counseling should directly address the misconception. A useful explanation is: “Collagen remodeling is stimulated by a safe and appropriate thermal dose, not by the highest number on the device. If the dose is too low, the treatment may be weak; if too high, tissue may be injured rather than rejuvenated.” Pain, redness, edema, and immediate tightness are not reliable endpoints for mature collagen remodeling, which evolves over weeks to months. Pain near the low‐to‐mid 40°C range may be a practical safety signal rather than a marker of superior efficacy [5, 6].

Clinically, RF energy prescription should integrate indication, device type, electrode configuration, tissue thickness, skin type, age, facial morphology, anatomical variation, prior procedures, patient tolerance, and manufacturer‐recommended parameters. Conventional RF may require repeated moderate passes, while RF microneedling should match needle depth and energy delivery to dermal or scar thickness. In every RF‐based platform, the operator should treat anatomy and tissue response rather than the marketing number.

Informed consent should state that stronger settings do not guarantee better collagen remodeling and may increase the risk of pain, burns, pigmentary change, fat loss, contour irregularity, scarring, dysesthesia, prolonged edema, or temporary motor nerve symptoms. Consent should also explain that rare nerve‐related events are usually described in case‐based literature and may include lower‐lip asymmetry from marginal mandibular branch irritation or brow asymmetry from frontal or temporal branch irritation [14, 15, 16, 17, 18, 24, 25]. Patients should understand that treatment parameters are selected according to anatomy, indication, device labeling, and safety margins rather than maximal output.

## 3 Conclusion

The clinical answer to “Is more energy better?” is no, not reliably. In energy‐based aesthetic practice, collagen remodeling is optimized by appropriate thermal dose rather than maximal device output. Preclinical RF data showing more favorable remodeling at 42 W than 73 W caution against energy maximalism, but such animal findings should not be interpreted as universal human treatment settings [1]. Safer aesthetic practice requires individualized, device‐specific, and anatomy‐based energy titration: enough controlled heating to stimulate remodeling, but not enough to increase avoidable pain, burns, fat injury, dysesthesia, scarring, nerve irritation, or loss of patient trust.

## Author Contributions

The author has read and approved the final manuscript. **Kyu‐Ho Yi:** conceptualization, writing – original draft preparation, writing – review and editing, visualization, supervision.

## Funding

The author has nothing to report.

## Ethics Statement

The author has nothing to report.

## Consent

The author has nothing to report.

## Conflicts of Interest

The author declares no conflicts of interest.

## Data Availability

The data that support the findings of this study are available from the corresponding author upon reasonable request.
